# Game-Based Interventions for Health Promotion in Older Adults: A Systematic Review of Protocols and ICF-Based Outcomes

**DOI:** 10.3390/healthcare14152313

**Published:** 2026-07-31

**Authors:** Susana Lopes, Raul Antunes, Cândida G. Silva, Marlene Rosa

**Affiliations:** 1 Doctoral School “Studii Salamantini”, University of Salamanca, 37008 Salamanca, Spain; 2 ciTechCare—Center for Innovation in Health Technologies and Care, Polytechnic University of Leiria, 2414-016 Leiria, Portugal; marlene.rosa@ipleiria.pt; 3 School of Education and Social Sciences, Polytechnic University of Leiria, 2411-901 Leiria, Portugal; raul.antunes@ipleiria.pt; 4 CIDESD—Research Center in Sport, Health and Human Development, 5000-801 Vila Real, Portugal; 5 School of Health Sciences, Polytechnic University of Leiria, 2411-901 Leiria, Portugal

**Keywords:** health promotion, older adults, game-based interventions, systematic review, ICF, serious games

## Abstract

**Highlights:**

**What are the main findings?**

- Statistically significant health-related outcomes were reported across multiple ICF domains in studies of game-based interventions.
- The included studies showed substantial variability in intervention structure, delivery format, and contextual characteristics, highlighting the multidimensional nature of game-based interventions in health promotion.

**What are the implications of the main findings?**

- The use of the ICF framework supports a multidimensional interpretation of health outcomes in game-based interventions.
- Future programs should focus on well-structured and context-sensitive intervention designs, with greater attention to long-term effects and the integration of multidimensional health outcomes.

**Abstract:**

Background/Objectives: Health promotion programs are essential for older adults, and game-based interventions have emerged as promising health-promotion approaches. This systematic review aimed to synthesize and characterize game-based intervention protocols and their effects on health promotion in older adults. Methods: Randomized controlled trials and quasi-experimental studies published between January 2014 and 31 May 2026, in English or Portuguese, with participants aged 60 years or older, were analyzed. Methodological quality and risk of bias were assessed using Joanna Briggs Institute tools. Health outcomes were classified according to the International Classification of Functioning, Disability and Health (ICF) domains. The PRISMA methodology was followed. Results: Twenty-nine studies were included, presenting methodological variability across several JBI domains. Health-related outcomes were identified across multiple ICF domains. Outcomes related to learning and health management showed statistically significant findings more frequently, whereas physical and cognitive outcomes were generally more heterogeneous. Long-term evidence remains limited. Conclusions: Game-based interventions appear to support health promotion in older adults across multiple domains of functioning. The findings suggest that differences in intervention structure, delivery format, and contextual characteristics may contribute to variability in reported outcomes. However, the heterogeneity of game-based interventions and outcome measures limits direct comparison across studies. Further research is needed to validate long-term effects and condition-specific applications.

## 1. Introduction

Population ageing is a major global public health challenge, with the number of people aged 60 years and older projected to increase substantially over the coming decades. According to the World Health Organization, this population is expected to nearly double, from 1 billion in 2020 to 2.1 billion by 2050 [1]. This demographic transition is accompanied by an increased burden of functional decline, often associated with musculoskeletal changes, cardiovascular disease, and cognitive dysfunction [2].

To prevent functional disability, health promotion requires proactive behaviours and multidimensional approaches. According to the World Health Organization [3], these programs are effective if they reinforce healthy behaviours and promote lifelong learning experiences. A focus on social interaction and personal empowerment has also been highlighted as potentially beneficial [4,5]. More innovative programs have adopted novel strategies, such as telehealth, digital strategies, and serious games [6,7]. Despite these recommendations, health promotion programs face limitations, including difficulties with long-term adherence, restricted access, and high demand. Participants’ lack of time and the difficulty in perceiving the real impact of health promotion programs are also limitations encountered [8,9]. Thus, there is a need for approaches that promote social support and improve awareness of health-related outcomes [10].

Game-based approaches have emerged as a potentially relevant strategy for health promotion, as they may combine social interaction with intrinsic motivation, essential factors for promoting the active participation of older adults. They also provide continuous feedback and progressive adaptations based on individual capabilities [11]. Hence, game-based approaches are potentially valuable for enhancing engagement and supporting participation in health promotion programs.

Despite the growing use of game-based interventions in health promotion, there is still a lack of structured synthesis regarding how these interventions are designed and applied across different health promotion contexts. Previous reviews have mainly focused on specific modalities, such as exergames or digital physical activity interventions [12,13], rather than on intervention structure and implementation characteristics across health promotion contexts. Moreover, the diversity of intervention approaches and health-related outcomes reported across studies highlights the need for integrative frameworks capable of supporting a multidimensional interpretation of game-based interventions in health promotion. In this context, the International Classification of Functioning, Disability and Health (ICF) provides a relevant framework to systematically classify and interpret health-related outcomes across different domains of functioning and participation.

In line with the multidimensional perspective of the International Classification of Functioning, Disability and Health (ICF), health promotion was considered to encompass interventions targeting physical, cognitive, psychological, behavioural, and social domains, provided that their primary objective was to enhance health, functioning, participation, or well-being rather than focusing exclusively on the treatment of a specific disease or health condition. Therefore, this systematic review aimed to synthesize and characterize game-based intervention protocols and their reported effects on health promotion in older adults, focusing on intervention characteristics, contextual variability, and targeted health domains using the ICF framework.

## 2. Materials and Methods

This systematic review was carried out in accordance with the Preferred Reporting Items for Systematic Reviews and Meta-Analyses (PRISMA) guidelines [14] and its protocol was registered with PROSPERO (CRD42024561816).

### 2.1. Research Strategy

A literature search was conducted on 31 May 2026 in three electronic databases (PubMed, Web of Science, and Scopus), covering studies published between January 2014 and 31 May 2026.

The selected publication period (2014–2026) aimed to capture contemporary game-based interventions, including the growing diversity of gamified and technology-supported health approaches applied in older adult populations over recent years.

The search strategy combined controlled vocabulary (e.g., MeSH terms, when applicable) and free-text terms related to aging, health promotion, and game-based interventions. Relevant keywords included “aging”, “older adults”, “health promotion”, “health literacy”, “game”, “serious games” and “gamification”. Boolean operators (“AND” and “OR”) were used to combine search terms, with search strategies adapted to the requirements of each database. Database-specific filters were applied for publication year (2014–2026) and language of publication (English or Portuguese). Only studies published in English and Portuguese were included due to feasibility considerations related to language proficiency and translation resources.

The complete database-specific search strategies are available in Supplementary Material S1.

### 2.2. Eligibility Criteria

Studies were included if they met the following criteria: (i) were published in English or Portuguese between January 2014 and 31 May 2026; (ii) were experimental or quasi-experimental studies that reported interventions involving participants aged 60 years or older, provided that health promotion outcomes were reported specifically for this age group; (iii) reported one or more outcome measures related to the impact of game-based interventions on health promotion (physical, cognitive, psychosocial, behavioral, or self-management-related measures associated with functioning, well-being, health literacy, or participation); and (iv) implemented interventions based on game-based strategies with a clearly described protocol.

Studies were excluded if they: (i) did not describe the game-based protocol; (ii) focused exclusively on usability or playability evaluations of programs with game-based components; or (iii) did not have the full text available, even after attempts to contact the authors. No minimum number of intervention sessions was established, as the review aimed not only to explore health-related outcomes, but also to characterize game-based intervention protocols across different implementation formats.

### 2.3. Study Selection Process

Following the database searches, the records were imported into a reference management database, and duplicates were excluded. The selection of studies was carried out independently by two reviewers to minimize selection bias and errors. A third reviewer was consulted to resolve potential disagreements.

The screening was carried out according to the predefined inclusion and exclusion criteria in two stages: first, title and abstract screening, followed by full-text assessment. The full texts of the studies were retrieved and evaluated independently by two reviewers (S.L. and M.R.). Disagreements were resolved through discussion and consensus. When consensus could not be reached, a third reviewer (R.A.) was consulted to resolve the disagreement.

### 2.4. Data Extraction

Data extraction was independently performed by two reviewers (S.L. and M.R.) and included information on bibliographic details (author and year of publication), study design, participants’ characteristics (sex and age), intervention characteristics (e.g., protocol, type of game, duration, and frequency) and health-related outcomes reported in the included studies. The characteristics of the included studies are presented in Table 1. Discrepancies between reviewers were resolved through discussion and consensus. When consensus could not be reached, a third reviewer (R.A.) was consulted.

All extracted health-related outcomes were subsequently classified according to the International Classification of Functioning, Disability and Health (ICF), following established linking rules [15], regardless of statistical significance. Each reported outcome was individually mapped to the corresponding ICF code. For each ICF code, the number of contributing studies, the total number of mapped outcomes, and the frequency and proportion of statistically significant and non-significant outcomes were summarized to provide a descriptive overview of the distribution of statistically significant and non-significant outcomes across the assessed health domains. Table 2 summarizes these data at the ICF-code level, including the number of contributing studies, the total number of mapped outcomes, and the distribution of statistically significant and non-significant outcomes. Supplementary Table S3 presents the outcome-level data, including assessment tools, time points, statistical significance, and corresponding ICF codes.

Outcomes were categorized according to the corresponding ICF component, including Body Functions (b), Activities and Participation (d), and Environmental Factors (e). ICF coding was independently performed by two reviewers (S.L. and M.R.) with experience in systematic reviews and ICF application. Disagreements regarding ICF classification were resolved through discussion and consensus. When the two reviewers were unable to reach consensus, a third reviewer (R.A.) was consulted to resolve the disagreement.

When missing data were identified, no attempts were made to contact the corresponding authors. Data extraction was restricted to information reported in the published full-text articles.

### 2.5. Quality Assessment and Risk of Bias

The methodological quality and risk of bias of the included studies were assessed using the Joanna Briggs Institute (JBI) checklists corresponding to randomized controlled trials (RCTs) [16] and quasi-experimental studies (QE) [17].

The JBI checklists comprise 13 items for randomized controlled trials and 9 items for quasi-experimental studies. Each item is classified as “yes”, “no”, “uncertain”, or “not applicable”. Two reviewers (S.L. and M.R.) independently assessed the included studies. Discrepancies in assessment were resolved by consensus or by consulting a third reviewer (R.A.).

Methodological quality was assessed using the JBI critical appraisal checklists, and item-level appraisal results are presented in Supplementary Tables S1 and S2.

Agreement between reviewers was evaluated using Cohen’s kappa score separately for title/abstract screening, full-text screening, data extraction, and study quality assessment.

## 3. Results

### 3.1. Selected Studies

**Figure 1:**
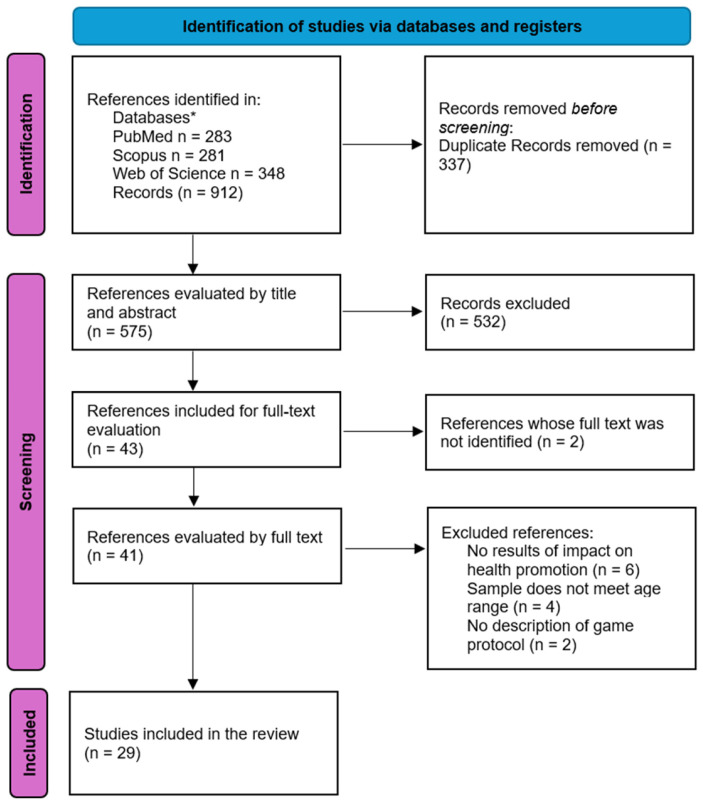
PRISMA flowchart of the study selection process (PRISMA 2020). * Consider, if feasible, reporting the number of records identified from each database or register searched rather than the total number across all databases/registers.

A total of 912 records were identified through database searches (PubMed: n = 283; Scopus: n = 281; Web of Science: n = 348). After removing 337 duplicate records, 575 references remained for title and abstract screening. Of these, 532 records were eliminated. Forty-three articles were selected for full-text retrieval; however, two full texts could not be accessed, resulting in 41 articles evaluated for eligibility. After full-text evaluation, 12 studies were excluded due to the absence of health promotion outcomes (n = 6), failure to meet age criteria (n = 4), or lack of description of the game-based protocol (n = 2). Consequently, 29 studies met the eligibility criteria and were included in this systematic review. The study selection process is presented in the PRISMA flowchart (Figure 1). The title and abstract screening resulted in 553 agreements and 22 disagreements between reviewers (Cohen’s κ = 0.72). The full-text screening resulted in 39 agreements and 2 disagreements between reviewers (Cohen’s κ = 0.86).

### 3.2. Quality Assessment and Risk of Bias

Methodological appraisal and risk of bias assessment were conducted for all included studies, with the detailed item-level assessment for each study provided in Supplementary Tables S1 and S2. The methodological quality assessment resulted in 28 agreements and 1 disagreement between reviewers (Cohen’s κ = 0.87).

The included studies presented methodological variability across several JBI domains. Among randomized controlled trials, the main methodological limitations were related to allocation concealment and blinding procedures, which were frequently classified as unclear or absent. Among quasi-experimental studies, common limitations included the lack of control over confounding factors and limited use of multiple pre/post measurements. Additionally, several studies presented limited follow-up assessment, particularly regarding long-term effects. These methodological limitations should be considered when interpreting the reported outcomes, particularly among quasi-experimental designs.

### 3.3. Characteristics of the Studies

The main characteristics of the studies are presented in Table 1. The data extraction process resulted in 27 agreements and 2 disagreements between reviewers (Cohen’s κ = 0.81). The included study designs were randomized controlled trials (n = 13) and quasi-experimental studies (n = 16). Sample sizes ranged from 15 to 372 participants, representing a total of 2007 participants. The interventions were implemented in various contexts, including community, care, residential, and home settings, and involved both digital and analog game-based approaches with substantial variability in duration, session frequency, and implementation formats.

### 3.4. Characterization of Participants

Participant characteristics are summarized in Table 1. Most studies presented a sample with more women than men, and there was one study with an exclusively female sample [18]. Two studies did not characterize participants by sex [19,20]. The mean age ranged from 68.2 years [18] to 89.9 years [21]. Most studies investigated samples of healthy older adults; however, five studies included specific health conditions, such as mild cognitive impairment (MCI) [22,23], sedentary lifestyle [24], functional limitations [24], and frailty [25].

### 3.5. Characterization of the Intervention in Experimental Group(s)

The characteristics of interventions in the experimental group(s) are also presented in Table 1. Overall, the included studies implemented both digital and analog game-based interventions. Digital games were used in 21 studies, whereas 8 studies employed analog game-based interventions. In five studies, the intervention protocol combined multiple games within the same program.

Across the included studies, intervention protocols frequently incorporated structuring strategies beyond gameplay itself. Several interventions implemented progressive increases in task difficulty, exercise intensity, or game complexity over time [21,26,27,28,29]. Some studies also adopted gradual reductions in supervision or promoted autonomous gameplay during later intervention phases [21,30,31]. Additionally, many protocols combined gameplay with health education, discussion moments, physical exercise, or reflective activities [22,28,29,32,33,34,35]. However, individualized adaptation strategies based on participant performance were inconsistently reported across studies.

### 3.6. Intervention Contexts

This review identified studies conducted across different settings, namely home, residential settings, care settings, and community settings (Table 1).

Studies conducted in the home setting included intervention protocols combining information sessions and initial game familiarization with subsequent autonomous implementation by participants. One of these studies [36] conducted preliminary sessions (four to five sessions) in a laboratory setting, followed by installation of the game in the home. Other studies reported follow-up visits throughout the intervention period. These protocols commonly included participants’ records of game usage time or satisfaction with the intervention.

The two studies carried out in residential structures [21,23] implemented group-based analog games. One intervention protocol was characterized by a progressive reduction in the number of supervised sessions, encouraging increasing autonomy in game use throughout the intervention program [21].

A total of eight studies were implemented in care settings. Most applied group-based interventions. Within this context, two studies reported adaptation of the game protocol to individual participant characteristics [25,37] and two studies combined different games within the same intervention protocol across the intervention period [18,38]. One study [37] combined game implementation with informational materials and discussion sessions on health-related topics. Notably, several interventions integrated physical activity with educational or informational components.

The community setting represented the context with the largest number of studies. One study [30] implemented a dual-format methodology in which participants played the game both individually and in groups at different points during the intervention, while another group played independently. Several studies reported protocol adaptations over time, including progressive inclusion of additional games [22,26] or increased physical challenge using weighted vests [39], as well as progressive increases in exercise intensity and task complexity [29]. Only one study reported individualized adaptation of the game protocol [27]. Additionally, several community-based interventions combined gameplay with other activities, such as health-related discussion moments [22], walking exercises [40] or provision of health education materials [28,31]. In this context, some protocols also encouraged autonomous activities between sessions [30,32] or competitive game modes [22,41], including pair-based competitive and cooperative gameplay formats [29].

Overall, interventions implemented in community and home settings more frequently incorporated autonomous gameplay, health education components, and progressive adaptations over time, whereas interventions conducted in residential and care settings more commonly relied on supervised and group-based formats. Digital game-based interventions predominated across studies; however, analog interventions were more frequently associated with group interaction and socially mediated gameplay formats. Despite these tendencies, substantial variability remained across intervention structures, participant profiles, comparator models, and implementation strategies.

### 3.7. Types of Intervention

All studies implementing analog games involved group-based interventions (Table 1). In contrast, studies using digital games were conducted in group formats in seven studies and individual formats in seven studies. Additionally, three studies included pair-based interventions, and one study reported a mixed intervention format, combining group and individual gameplay.

Regarding studies implementing analog games in group settings, group sizes ranged from two participants to 20–30 participants. In studies implementing digital games, group sizes ranged from two to four participants to eight participants.

### 3.8. Game Implementation Dosage

The duration of the intervention programs ranged from a single session to 24 weeks, with most protocols lasting 12 weeks (Table 1). Three included studies adopted a single-session intervention format.

Session frequency varied across studies, with once-weekly sessions being the most reported frequency. Higher frequencies were also reported, ranging from daily sessions to five sessions per week, as well as variable session frequencies throughout the intervention period.

Session duration ranged from 20 min to 120 min, with 60 min being the most frequently reported session length. Several studies reported a range of session durations, rather than a fixed duration per session.

**Table 1:** Characterization of the studies included (n = 29).

Author (Year)	Study	Sample	Characterization of the Intervention in the EG			
			Game	Dosage	IC	IT
Bao et al., 2025 [42]	QE	n = 48 (healthy) 40 F; 8 M Age: 71 (6.68) EG1: n = 10 EG2: n = 8 EG3: n = 19 CG: n = 11	Nintendo Wii Fit	4 wks 1×/wks 30′/session	Care Structure	Digital Game Individual (EG1, EG2) Pairs (EG3)
Belchior et al., 2019 [36]	RCT	n = 54 (healthy) 34 F; 20 M Age: 73.2 (5.5) EG1: n = 17 EG2: n = 19 CG: n = 18	EG1: CrazyTaxi EG2: InSight	12 wks 5×/wk 60′/session	Home	Digital Game Individual
Berman et al., 2020 [32]	QE	n = 123 (healthy) 104 F; 19 M Age: 74.4 (8.4)	Age-Tastic Health Education Game	8 wks 1×/wk 60′/session	Community	Analog Game Group (6 to 15 players)
Chao et al., 2015 [37]	QE	n = 32 (healthy) EG: n = 16 11 F; 5 M Age: 86.63 (4.18) CG: n = 16 13 F; 3 M Age: 83.75 (8.04)	Nintendo Wii Fit	4 wks 2×/wk 60′/session (30′/participant)	Care Structure	Digital Game Pairs
Clifford et al., 2014 [19]	QE	n = 18 (healthy) Age: ≥60	Knowledge Game	1 session 45′ to 60′	Community	Analog Game Group
Crandall et al., 2019 [34]	RCT	n = 105 (healthy) EG: n = 60 51 F; 9 M Age: 73.59 (7.87) CG: n = 45 39 F; 6 M Age: 73.22 (7.79)	Bingocize	10 wks 2×/wk 60′/session	Care Structure	Digital Game Group
Dispennette et al., 2019 [35]	RCT	n = 36 (healthy) EG: n = 19 17 F; 2 M Age: 70.89 (4.92) CG: n = 17 15 F; 2 M Age: 76.88 (7.77)	Bingocize	12 wks 2×/wk 45′ to 60′/session	Community	Digital Game Group
Hughes et al., 2014 [22]	RCT	n = 20 (MCI) EG: n = 10 8 F; 2 M Age: 78.5 (7.1) CG: n = 10; 6 F; 4 M Age: 76.2 (4.3)	Nintendo Wii Fit	24 wks 1×/wk 90′/session	Community	Digital Game Group (3 to 4 players)
Huang et al. (2025) [29]	RCT	n = 30 (healthy) EG: n = 15; 13 F; 2 M Age: 67.8 (3.9) CG: n = 15; 13 F; 2 M Age: 67.7 (4.4)	Stampede exergame-based mat training	10 wks; 2×/wk; 70′/session	Community	Digital Game Pairs
Hsu I et al., 2023 [33]	QE	n = 233 (healthy) 192 F; 41 M Age: 71.1 (7.1)	Kaban Game	1 session 120′	Community	Analog Game Group (20 to 30 players)
Iizuka et al., 2019 [30]	RCT	n = 72 (healthy) EG1: n = 25 18 F; 7 M Age: 76.8 (5.4) EG2: n = 22 16 F; 6 M Age: 77.0 (3.5) CG: n = 25 20 F; 5 M Age: 76.5 (4.6)	GO/GO quest Kifu-narabe	12 wks EG1: 1×/wk 60′/session EG2: Autonomous	Community	Digital Game EG1: individual + group EG2: individual
Kwan et al., 2024 [43]	RCT	n = 25 (cognitive frailty) 17 F; 8 M Age: ≥60 EG: n = 13 CG: n = 12	Gamified Home-Based Cognitive-Nutritional Training Programme: Cognitive Training Game Nutrition Revision Game	EG: 12 wks (4 + 8) 5×/wk 30′/session CG: Health education	Home	Digital Game Individual
Lee et al., 2014 [39]	QE	n = 82 (healthy) 58 F; 24 M Age: 75.2 (6.6) EG: n = 42 CG: n = 40	Nintendo Wii Fit	10 wks 3×/wk 45′/session	Community	Digital Game Group
Lee et al., 2020 [38]	QE	n = 150 (healthy) EG: n = 75 62 F;13 M Age: 76.99 (8.08) CG: n = 75 54 F; 51 M Age: 76.69 (7.86)	Zicke Zacke HuhnerKacke Hisss Noah’s ark Speed cups Rummikub	12 wks 1×/wk 120′/session	Care Structure	Analog Game Group (5 to 6 players)
Lee et al., 2015 [18]	QE	n = 15 (healthy) 15 F Age: ≥65	Paldokangsan1/2 Games with floor platform and hand control	12 wks 2×/wk	Care Structure	Digital Game Individual
Martins et al., 2020 [44]	QE	n = 35 (healthy) EG: n = 18 13 F; 3 M Age: 83.06 (8.52) CG: n = 16 13 F; 3 M Age: 84.88 (7.27)	FallSensing Exergames (3 minigames)	8 wks 3×/wk 20′/session	Care Structure	Digital Game Group (Up to 3 players)
Mouton et al., 2017 [21]	QE	n = 21 (healthy) EG: n = 10 6F; 4M Age: 82.5 CG: n= 11 8F; 3M Age: 89.9	Giant floor board game: 24 squares and a walking track	4 wks wk1—4× wk2—3× wk3—2× wk4—1× 30′ to 60′/session	Residential Structure	Analog Game Group (Minimum 2 players)
Nonino et al., 2018 [20]	RCT	n = 24 (sedentary) EG n = 12 Age: 71.5 (5.7) CG n = 12 Age: 68.2 (4.5)	Nintendo Wii Sports (bowling)	8 wks Autonomous	Home	Digital Game Individual
Onishi et al., 2022 [41]	QE	n = 25 (healthy) 16 F; 9 M Age: 75 (8)	PlayStation 4-Gran Turismo Sport	1 session 25′ to 30′	Community	Digital Game Pairs
Phirom et al., 2020 [27]	RCT	n = 40 (healthy) EG: n = 20 17 F; 3 M Age: 70.21 (4.18) CG: n = 20 16 F; 4 M Age: 69.4 (3.38)	Fruits Hunter Where am I? Whack a mole Sky fall Crossing poison river	12 wks 3×/wk 60′/session	Community	Digital Game with floor projection Individual
Roopchand-Martin et al., 2015 [26]	QE	n = 33 (healthy) 26 F; 7 M Age: 70.25 (6.69)	Nintendo Wii Fit	6 wks 2×/wk 30′/session	Community	Digital Game
Sayar et al., 2023 [40]	RCT	n = 40 (healthy) 23 F; 17 M Age: 69.4 (3.38)	Kinect Adventur! Your Shape Fitness Evolved 2012	2 wks 1×/wk 30′/session	Community	Digital Game
Shake et al., 2018 [45]	RCT	n = 105 (healthy) EG: n = 60 51 F; 9 M Age: 73.59 (7.87) CG: n = 45 39 F; 6 M; Age: 73.2 (7.79)	Bingocize	12 wks 2×/wk 60′/session	Care Structure	Digital Game Group
Strand et al., 2014 [31]	QE	n = 46 (healthy) 40 F; 6 M Age: ≥60	Nintendo Wii EA Sports Active	24 wks Stage 1: 8wks 2×/wk 13′ to 23′/session Stage 2: 16 wks 2×/month Autonomous	Community	Digital Game Individual
Szanton et al., 2016 [24]	QE	n = 21 (with functional limitations) 16 F; 6 M Age: 73.6 (7.2)	Nintendo Wii Fit-Game based on Tai Chi principles	4 wks 1×/day	Home	Digital Game Individual
Tsai et al., 2024 [46]	QE	n = 85 (healthy) EG: n = 43 27 F; 16 M Age: 71.2 CG: n = 42 29 F; 13 M Age: 71.4	Bone Prevention Board Game	4 wks 1×/wk 90′/session	Care Structure	Analog Game Group (6 to 7 players)
Tsai et al., 2025 [28]	QE	n = 49 (healthy) 37 F; 12 M Age: 73.7 (8.0)	Stable Sugar Winner board game	4wks 1×/wks 90′/session	Community	Analog Game Group (5 to 6 players)
Verghese et al., 2021 [25]	RCT	n = 372 (frail) EG: n = 186 135 F; 51 M Age: 76.9 (5.7) CG: n = 186 136 F; 50 M Age: 77.1 (5.6)	33 games-CogniFit	8 wks 3×/wk 50′ session	Care Structure	Digital Game Group (Up to 8 players)
Yang et al., 2022 [23]	RCT	n = 68 (MCI) EG: n = 34 19 F; 15 M Age: 78.74 (5.79) CG: n = 34 19 F; 15 M Age: 78 (6.85)	Sammy Robot	12 wks 1×/wk 120′ session	Residential Structure	Analog Game using robotics Group

Age presented in years. IC—Intervention Context; IT—Intervention Type; RCT—Randomized Controlled Trial; QE—Quasi-Experimental Study; F—Female; M—Male; EG—Experimental Group; CG—Control Group; wk(s)—week(s); ′—Minutes; MCI—Mild Cognitive Impairment.

### 3.9. Assessment Instruments and Impact Measures

Approximately 80 different assessment instruments were used in the included studies (Supplementary Table S3). Within the physical domain, the Timed Up and Go (TUG), including the dual-task TUG (n = 6), and the Short Physical Performance Battery (n = 4) were the most frequently used assessment tools. The 30 s sit-to-stand test, the Berg Balance Scale, the Falls Efficacy Scale, the Trail Making Test, and handgrip strength were also used. In the psychological and cognitive domains, some of the instruments used in more than one study were the Positive and Negative Affect Scale (PANAS), the Geriatric Depression Scale (GDS), and the Mini-Mental State Examination (MMSE). Several questionnaires were used in the studies, most of which were developed by the researchers specifically for their studies. Most questionnaires aimed to assess knowledge about health; only one study aimed to assess lifestyle changes. Finally, in-game performance, assessed through scores obtained, was used in two studies.

### 3.10. Health-Related Outcomes According to the ICF

The classification of health-related outcomes according to the ICF resulted in the identification of 49 codes, corresponding to 28 codes for Body Functions (b), 20 for Activities and Participation (d), and one for Environmental Factors (e). Table 2 presents, for each ICF code, the number of studies in which it was evaluated, the total number of outcomes mapped, and the distribution between statistically significant and non-significant results. Detailed outcome-level information for each study, including the assessment instrument, the time of assessment, the statistical significance, and the respective ICF code, is presented in Supplementary Table S3.

The ICF codes most frequently assessed across the included studies were each assessed in 9 to 14 of the 29 included studies: Walking (d450), Looking after one’s health (d570), Emotional functions (b152), Muscle power functions (b730), Vestibular balance functions (b2351), and Learning and applying knowledge, other specified (d198).

When the proportion of statistically significant and non-significant outcomes mapped to each ICF code was considered, different proportions were observed among the assessed health domains.

Among the Body Functions codes related to physical function, Control of voluntary movement functions (b760; 79.2% statistically significant outcomes) and Muscle endurance functions (b740; 62.5%) showed a predominance of statistically significant outcomes. In contrast, Muscle power functions (b730; 47.4% statistically significant outcomes), Vestibular balance functions (b2351; 50%), and Gait pattern functions (b770; 57.1%) showed a more balanced distribution between statistically significant and non-significant outcomes.

**Table 2:** Summary of health-related outcomes by ICF code.

ICF Component	ICF Code	ICF Description	Studies Assessing the Code (n)	Outcomes Assessed (n)	Significant Outcomes, n (%) *	Non-Significant Outcomes, n (%) *
Body Functions						
	b110	Consciousness functions	4	4	2 (50.0)	2 (50.0)
	b114	Orientation functions	1	1	0 (0.0)	1 (100.0)
	b126	Temperament and personality functions	3	3	3 (100.0)	0 (0.0)
	b1300	Energy level	3	4	3 (75.0)	1 (25.0)
	b140	Attention functions	7	8	4 (50.0)	4 (50.0)
	b1402	Dividing attention	1	1	1 (100.0)	0 (0.0)
	b144	Memory functions	6	15	4 (26.7)	11 (73.3)
	b147	Psychomotor functions	1	1	0 (0.0)	1 (100.0)
	b152	Emotional functions	11	24	12 (50.0)	12 (50.0)
	b1561	Visual perception	1	2	2 (100.0)	0 (0.0)
	b1565	Visuospatial perception	1	4	0 (0.0)	4 (100.0)
	b164	Higher-level cognitive functions	5	9	3 (33.3)	6 (66.7)
	b167	Mental functions of language	5	8	1 (12.5)	7 (87.5)
	b210	Seeing functions	1	2	1 (50.0)	1 (50.0)
	b2351	Vestibular balance functions	9	38	19 (50.0)	19 (50.0)
	b260	Proprioceptive function	1	2	1 (50.0)	1 (50.0)
	b280	Sensation of pain	2	2	1 (50.0)	1 (50.0)
	b4100	Heart rate	4	6	5 (83.3)	1 (16.7)
	b420	Blood pressure functions	3	7	4 (57.1)	3 (42.9)
	b455	Exercise tolerance functions	3	9	3 (33.3)	6 (66.7)
	b540	General metabolic functions	1	1	1 (100.0)	0 (0.0)
	b710	Mobility of joint functions	2	4	1 (25.0)	3 (75.0)
	b7101	Mobility of several joints	1	4	3 (75.0)	1 (25.0)
	b730	Muscle power functions	10	19	9 (47.4)	10 (52.6)
	b7300	Power of isolated muscles and muscle groups	1	3	1 (33.3)	2 (66.7)
	b740	Muscle endurance functions	6	8	5 (62.5)	3 (37.5)
	b750	Motor reflex functions	1	2	2 (100.0)	0 (0.0)
	b760	Control of voluntary movement functions	3	24	19 (79.2)	5 (20.8)
	b770	Gait pattern functions	6	7	4 (57.1)	3 (42.9)
**Activities and Participation**						
	d6	Domestic life	1	2	2 (100.0)	0 (0.0)
	d7	Interpersonal interactions and relationships	4	4	2 (50.0)	2 (50.0)
	d9	Community, social and civic life	2	3	3 (100.0)	0 (0.0)
	d155	Acquiring skills	2	3	3 (100.0)	0 (0.0)
	d198	Learning and applying knowledge, other specified	9	20	13 (65.0)	7 (35.0)
	d230	Carrying out daily routine	3	3	1 (33.3)	2 (66.7)
	d360	Using communication devices and techniques	1	1	1 (100.0)	0 (0.0)
	d415	Maintaining a body position	1	2	0 (0.0)	2 (100.0)
	d420	Transferring oneself	6	7	4 (57.1)	3 (42.9)
	d450	Walking	14	30	13 (43.3)	17 (56.7)
	d470	Using transportation	1	1	0 (0.0)	1 (100.0)
	d510	Washing oneself	2	2	1 (50.0)	1 (50.0)
	d570	Looking after one’s health	12	16	11 (68.8)	5 (31.2)
	d6200	Shopping	1	1	0 (0.0)	1 (100.0)
	d630	Preparing meals	1	1	0 (0.0)	1 (100.0)
	d640	Doing housework	1	2	0 (0.0)	2 (100.0)
	d850	Remunerative employment	2	2	1 (50.0)	1 (50.0)
	d860	Basic economic transactions	1	1	0 (0.0)	1 (100.0)
	d920	Recreation and leisure	1	1	1 (100.0)	0 (0.0)
	d9200	Play	2	3	3 (100.0)	0 (0.0)
**Environmental Factors**						
	e5	Services, systems and policies	1	1	0 (0.0)	1 (100.0)

* Percentages were calculated based on the total number of outcomes mapped to each ICF code.

Within Body Functions, the codes related to cognition and mental functions showed distinct distributions. While Attention functions (b140; 50.0% statistically significant outcomes) and Emotional functions (b152; 50%) showed similar proportions of statistically significant and non-significant outcomes, Memory functions (b144; 73.3% non-significant outcomes), Higher-level cognitive functions (b164; 66.7%), and Mental functions of language (b167) showed a predominance of non-significant results.

In the Activities and Participation component, the codes related to knowledge and health-related behaviors, namely, Looking after one’s health (d570; 68.8% statistically significant outcomes) and Learning and applying knowledge, other specified (d198; 65%), showed a predominance of statistically significant outcomes. In contrast, Walking (d450; 43.3% statistically significant and 56.7% non-significant outcomes) showed a balanced distribution between statistically significant and non-significant outcomes.

The Environmental Factors component was represented by only one code (e5), identified in a single study [29], and no statistically significant results were observed.

Regarding long-term follow-up, only three studies included this assessment [21,31,36]. Exclusively statistically significant follow-up outcomes were reported for attention functions (b140), visual perception (b1561), general metabolic functions (b540), acquiring skills (d155), playing (d9200), and carrying out daily routine (d230). Exclusively non-significant follow-up outcomes were identified for memory functions (b144), walking (d450), transferring oneself (d420), gait pattern functions (b770), and visual perception (b1565). Both statistically significant and non-significant follow-up outcomes were reported for emotional functions (b152), vestibular balance functions (b2351), muscle power functions (b7300), and looking after one’s health (d570). Among these, emotional functions (b152) and vestibular balance functions (b2351) showed a balanced distribution of statistically significant and non-significant outcomes, whereas looking after one’s health (d570) showed a predominance of statistically significant outcomes and muscle power functions (b7300) showed a predominance of non-significant outcomes.

## 4. Discussion

This systematic review synthesized evidence on game-based intervention protocols and their reported effects on health promotion in older adults, highlighting the multidimensional and context-dependent nature of these interventions.

Overall, the included studies describe both digital and analog games as multidimensional intervention approaches targeting physical, cognitive, psychosocial, and participation-related domains of health in older adults. However, substantial variability across intervention structures, implementation contexts, participant profiles, and outcome measures limits comparability across studies and restricts stronger conclusions regarding intervention effectiveness. Importantly, the heterogeneity identified across studies should not be interpreted solely as a methodological limitation but may also reflect the complexity and contextual nature of health promotion interventions in older adults. Differences in supervision, autonomy, progression strategies, integration of health education components, and social dynamics suggest that the effects of game-based interventions may depend not only on the type of game used, but also on how gameplay is structured and integrated within broader intervention programs.

Digital games predominated over analog formats in health promotion interventions for older adults. This predominance may be associated with the increasing accessibility and acceptance of digital technologies among older adults, rather than necessarily reflecting superiority over analog approaches [47]. Previous evidence has also described the increasing acceptance of digital technologies and improved access to digital devices among older adults, corroborating this trend [48]. However, the marked heterogeneity in intervention design, implementation strategies, and reporting standards makes it difficult to determine whether these differences are attributable to the technological format itself or to broader characteristics of the intervention protocols.

On the other hand, digital games allow adjustments in the level of difficulty and personalized feedback, which may support adherence and participant engagement [49]. These characteristics may partly explain why, in the studies included in this review, digital interventions were more frequently associated with cognitive stimulation, autonomous gameplay, and opportunities for participation outside supervised settings.

However, despite the recognized importance of personalization in health promotion interventions, only a limited number of studies clearly described adaptation strategies based on participant characteristics or performance, highlighting the underuse of this potential [13]. This lack of detailed reporting regarding progression criteria, adaptation processes, and therapeutic framing limits understanding of the mechanisms through which game-based interventions may influence health-related outcomes. Additionally, the methodological limitations identified across several quasi-experimental studies, particularly regarding confounding factors, blinding procedures, sample size, and limited follow-up assessment, should be considered when interpreting the reported intervention effects. The multidimensional outcomes identified across studies may reflect the interaction of multiple active components beyond gameplay itself, including physical activity, cognitive engagement, health education, feedback mechanisms, and social interaction [50]. However, the relative contribution of these components remains insufficiently explored across existing interventions.

Although digital games predominated across the included studies, differences between digital and analog approaches appeared to extend beyond technological format alone. Analog games were more frequently implemented in group-based community or care settings and were commonly associated with health education, discussion moments, and social interaction components [19,28,32,33,38,46]. In contrast, digital interventions more frequently included autonomous or semi-autonomous gameplay, home-based implementation, exergaming, or cognitive training approaches [18,24,25,36,45]. These differences suggest that digital and analog games may reflect distinct implementation models and therapeutic strategies within health promotion interventions for older adults, rather than simply representing alternative technological formats.

Group implementation was common in the interventions analyzed, being present in all interventions using analog games [19,21,23,28,32,33,38,46] but also predominant in digital interventions [22,25,34,35,39,44,45]. These findings suggest that social interaction may play a relevant role within game-based health promotion interventions, particularly regarding motivation, engagement, and reduction in loneliness [51]. Social interaction may also contribute to adherence, emotional engagement, and participation-related outcomes, particularly in interventions implemented within community and care settings [52].

Mixed approaches that combine group and individual intervention formats, such as those described in Iizuka et al. [30], remain underexplored, despite their potential for balancing social engagement with individualized practice. However, the limited number of studies restricts conclusions regarding effectiveness, highlighting the need for further research in this area. Similarly, autonomous or semi-autonomous gameplay was mainly identified in home-based interventions [20,24,36,43] and in some community-based protocols, particularly during later intervention phases [31]. Previous evidence suggests that exergame interventions, including autonomous implementations, may contribute to improvements in functional and mental health parameters in older adults [43,53].

Community and group-based interventions more frequently reported outcomes related to emotional functions, participation, learning, and health management domains, whereas home-based interventions appeared more frequently associated with autonomous gameplay, cognitive stimulation, and continuity of participation outside supervised settings. Although these observations do not allow conclusions regarding comparative effectiveness, they suggest that intervention context and implementation structure may influence the outcomes most frequently reported, particularly those related to emotional functions, participation, learning, and health management domains. Importantly, the present review was not designed to formally compare the effectiveness of different intervention formats or implementation models, and these observations should be interpreted cautiously and descriptively.

Taken together, these findings suggest that the balance between supervision, autonomy, and social interaction may represent an important consideration when designing future game-based health promotion interventions for older adults.

On the other hand, motivational mechanisms, such as competition, were rarely implemented, with only two studies mentioning their use [22,41]. Competitive game elements have previously been described as possible strategies to encourage participation and engagement [54]. However, previous evidence highlights the distinction between social competition and self-competition in older adults, suggesting that competition against others may contribute to frustration or demotivation, whereas self-competition strategies may better support intrinsic motivation and sustained engagement over time [55]. Importantly, the studies included in this review focused exclusively on social competition approaches, with no explicit description of self-competition strategies, highlighting a potentially underexplored mechanism for supporting intrinsic motivation in older adults.

Health education protocols were integrated into several interventions [19,28,32,33,34,35,46], with some programs combining health education with physical activity [34,35]. Previous reviews have highlighted the potential benefits of combining physical exercise and health education strategies, particularly when supported by digital approaches that may increase accessibility and facilitate participation among older adults with mobility limitations or restricted access to in-person services [56]. Although several studies included in this review reported positive findings associated with combined health education and physical exercise strategies, limited evidence was found regarding the isolated contribution of health education components, particularly concerning health literacy-related outcomes. Some included studies involved single-session interventions, which may limit the interpretation of findings related to sustained health promotion over time. However, these studies were retained because the review also aimed to characterize intervention structures and identify exploratory health-related outcomes associated with emerging game-based approaches. Although single-session interventions were intentionally retained to provide a comprehensive characterization of game-based intervention protocols, isolated activities may not reflect the sustained behavioural changes typically associated with health promotion programmes. Consequently, the inclusion of these studies should be considered when interpreting the overall findings of this review regarding game-based interventions for health promotion.

### 4.1. ICF-Based Interpretation of Health-Related Outcomes

The ICF-based synthesis identified a broad range of health-related outcomes across the different ICF components, particularly Body Functions and Activities and Participation, consistent with the multidimensional nature of game-based interventions for older adults. However, the distribution of statistically significant and non-significant outcomes differed considerably across ICF codes and health domains. Importantly, the ICF codes most frequently assessed were not necessarily those presenting the highest proportion of statistically significant outcomes.

Among Activities and Participation, outcomes related to learning and health management were more frequently reported as statistically significant. Learning and applying knowledge, other specified (d198), was primarily assessed in interventions explicitly designed to improve health-related knowledge, including several health education topics, such as depression, antibiotic use, falls, osteoporosis, and diabetes prevention [19,28,32,33,34,35,46]. Similarly, looking after one’s health (d570) was frequently associated with interventions targeting physical activity, dietary behaviours, preventive practices, exercise self-efficacy, and health self-management [18,21,23,28,29,31,34,37,42,43,44,46]. The higher proportion of reported statistically significant outcomes for these ICF codes may therefore partly reflect the direct alignment between the intervention objectives and the outcomes used to evaluate them. These findings also reinforce the inclusion of educational, behavioural, and self-management domains within health promotion, distinguishing these interventions from programmes focused exclusively on rehabilitation or the recovery of specific functional deficits [6,8].

In contrast, outcomes related to physical function showed greater variability. This variability may partly reflect the substantial heterogeneity identified among the interventions, including differences in game type, intervention duration, training frequency, supervision, progression strategies, and the combination of physical, cognitive, and educational components. Previous reviews of exergames and digital physical activity interventions have similarly reported considerable heterogeneity in intervention design, training dose, and physical outcomes among older adults [12,13,53,54].

The variability observed for walking (d450) may also partly reflect the diversity of assessment methods mapped to this ICF code. Although all measures were related to mobility, they ranged from objective assessments of gait performance and functional mobility to endurance and self-reported physical activity [18,20,21,22,24,25,27,29,35,37,39,43,44,45]. Consequently, aggregation within the same ICF code facilitates functional comparison but does not eliminate the clinical and methodological differences between the outcomes assessed.

A similar pattern was observed for cognitive and mental functions, suggesting that cognitive outcomes were not equally responsive to game-based interventions. While memory, higher-level cognitive functions and mental functions of language were predominantly associated with non-significant findings, attention and emotional functions showed more balanced results. Several studies reporting statistically significant cognitive outcomes assessed functions closely related to the cognitive components incorporated into the intervention protocols, such as visual attention, executive processing, and game-specific cognitive skills [27,30,36]. In contrast, studies evaluating broader cognitive domains or overall cognitive performance more frequently reported mixed or predominantly non-significant findings [22,23,25,43,45]. These findings suggest that statistically significant improvements were more consistently reported for cognitive functions closely related to those addressed during the intervention, whereas broader cognitive domains showed less consistent results.

In contrast, Environmental Factors remained substantially underrepresented in the studies included in this systematic review, despite the fact that many interventions were implemented in homes, community settings, care facilities and residential structures. In the complementary literature related to healthy ageing frameworks, functional ability results from the interaction between individual capacities and the surrounding environment, including social support, accessibility, services, and opportunities for participation [1,3,50]. Therefore, the limited evaluation of Environmental Factors restricts understanding of how contextual facilitators and barriers may influence intervention participation, implementation, and sustainability.

Follow-up assessments were reported in only three studies [21,31,36], limiting conclusions regarding the maintenance of intervention effects. Although sustained statistically significant improvements were observed in selected cognitive, emotional, behavioural and functional outcomes, other mobility, muscle strength and cognitive outcomes remained non-significant at follow-up. The small number of studies, together with differences in intervention duration, follow-up periods and assessed outcomes, limits stronger conclusions regarding the maintenance and transfer of intervention effects. This is consistent with previous evidence indicating that the long-term effects of physical activity interventions may vary according to the characteristics of the intervention, the outcomes assessed and the duration of follow-up [57].

Similarly, the inclusion of three single-session studies [19,33,41] had little influence on the overall ICF synthesis. With the exception of energy level (b1300), all ICF codes identified in these studies were also represented in multi-session interventions, and excluding the single-session studies would not substantially alter the overall distribution of statistically significant and non-significant outcomes. Although single-session interventions cannot provide evidence regarding sustained health promotion effects, their inclusion allowed the characterization of emerging intervention formats and immediate outcomes related to health knowledge, emotional responses and physiological parameters, while acknowledging that these findings should not be interpreted as evidence of sustained health promotion effects.

### 4.2. Integrated Interpretation and Implications

The results of this review suggest that game-based interventions may represent a relevant multidimensional approach for health promotion in older adults, particularly regarding Body Functions and Activities and Participation domains. The studies analyzed suggest that intervention structure, implementation characteristics, and contextual factors may contribute to variability in reported outcomes across game-based interventions.

Although there is a predominance of group interventions across the included studies, mixed and autonomous approaches are beginning to emerge, highlighting the diversity of possible implementation models in game-based health promotion interventions.

Community and group-based interventions frequently combined gameplay with physical exercise, health education, discussion moments, and social interaction and were more commonly associated with outcomes related to participation, emotional functions, learning, and health management domains. In contrast, home-based and more autonomous interventions more frequently emphasized cognitive stimulation, exergaming, continuity of participation outside supervised settings, and self-management-related components.

The differences observed between digital and analog interventions appear to go beyond the technological format itself, potentially reflecting distinct models of therapeutic integration and implementation priorities in health promotion programs for older adults. Additionally, games were frequently integrated with physical exercise, cognitive stimulation, health education, or social interaction components and were associated with multidimensional outcomes across different ICF domains. These findings suggest that the reported effects may emerge from the interaction of multiple intervention components rather than from gameplay alone.

However, explanations regarding game-based mechanisms, progression criteria, adaptation processes, and therapeutic framing remain limited across studies. Overall, these findings reinforce the importance of better conceptual and methodological descriptions of game-based interventions, particularly regarding how gameplay is intentionally integrated within broader health promotion strategies for older adults.

### 4.3. Limitations and Future Directions

The findings identified in this review should be interpreted in light of several important limitations. Most included studies involved samples of healthy older adults and predominantly female participants, which may limit transferability of findings across broader and more vulnerable older adult populations. Additionally, considerable heterogeneity was observed across participant profiles, intervention structures, implementation contexts, and assessment instruments, limiting comparability between studies and interpretation of reported outcomes. Although this heterogeneity reflects, in part, the complexity and contextual nature of health promotion interventions, it also restricts stronger conclusions regarding intervention effectiveness.

The limited assessment of environmental and contextual factors across the included studies further limits understanding of how implementation settings, social support, and real-world conditions may influence participation and intervention effects. Moreover, the limited number of longitudinal studies restricts stronger conclusions regarding the persistence of intervention effects over time. Some included studies also evaluated single-session interventions, which contribute to the characterization of emerging game-based approaches. However, their inclusion limits the interpretation of findings related to sustained health promotion outcomes and should be considered when interpreting the overall findings of this review.

Additional limitations related to the methodological procedures of this systematic review should also be acknowledged. No contact was established with study authors to obtain missing information. Data extraction was therefore restricted to information reported in the published full-text articles. Consequently, incomplete reporting within the original publications may have limited the completeness of the extracted information, including intervention descriptions, outcome reporting, and data synthesis. Therefore, some intervention characteristics and health-related outcomes may not have been fully captured in this review. Furthermore, although ICF classification followed established linking guidelines, the interpretation and linking of outcomes to ICF categories involve a degree of subjectivity, which may have influenced the classification of some outcomes.

Furthermore, although the ICF-based synthesis included both statistically significant and non-significant outcomes, several methodological aspects should be considered when interpreting these findings. The ICF provides a standardized framework for classifying conceptually related outcomes across different assessment instruments. Consequently, different instruments assessing the same functional domain may be mapped to the same ICF code despite capturing distinct aspects of that domain. Moreover, individual studies could contribute multiple outcomes to the same ICF code, potentially increasing the number of outcomes contributing to specific ICF codes. Consequently, the frequency of ICF codes presented in this review should be interpreted as a descriptive synthesis of the reported outcomes rather than as a quantitative estimate of intervention effectiveness. Supplementary Table S3 was included to ensure transparency by providing complete outcome-level mapping, including assessment instruments, assessment time points, statistical significance, and the corresponding ICF codes.

These limitations suggest that future research should move beyond evaluating games as isolated interventions, prioritizing clearer conceptual descriptions, multidimensional intervention models, and context-adapted implementation strategies. Greater consistency in intervention reporting, implementation protocols, and outcome assessment would also facilitate comparisons across studies and strengthen the evidence base for game-based health promotion interventions. Future studies should also explore how game-based interventions can be better adapted to the complexity of aging processes and to the transfer of gains into daily life contexts.

## 5. Conclusions

This systematic review found that game-based interventions have been applied to address a broad range of health-related outcomes in older adults, spanning multiple domains of functioning within the ICF framework. However, the consistency of the reported evidence varied considerably across the health domains assessed.

The findings of this review suggest that the reported effects may depend not only on the type of games used, but also on how interventions are designed, adapted, and implemented within broader health promotion approaches.

Although digital games predominated across the included studies, differences between digital and analog interventions appeared to extend beyond technological format alone, reflecting different implementation contexts and intervention characteristics.

Although several health domains showed predominantly statistically significant outcomes, the substantial methodological heterogeneity, variability in intervention structures and assessment methods, the inclusion of some single-session interventions, and limited longitudinal evidence restrict stronger conclusions regarding long-term intervention effects and the overall interpretation of the findings.

Future research should prioritize clearer conceptual and methodological descriptions of game-based interventions, greater consideration of contextual and environmental factors, and longitudinal studies capable of clarifying how different intervention models may influence multidimensional health-related outcomes in older adults. Greater consistency in intervention reporting and outcome assessment would also facilitate comparisons across studies and strengthen the evidence base for game-based health promotion interventions.

## Data Availability

No new data were created or analyzed in this study. Data sharing is not applicable.

## Supplementary Materials

The following supporting information can be downloaded at: https://www.mdpi.com/article/10.3390/healthcare14152313/s1. Supplementary Material S1: Database-specific search strategy; Supplementary Table S1: Methodological quality assessment of randomized controlled trials using the Joanna Briggs Institute (JBI) critical appraisal checklist; Supplementary Table S2: Methodological quality assessment of quasi-experimental studies using the Joanna Briggs Institute (JBI) critical appraisal checklist; Supplementary Table S3: Outcome-level characteristics of health-related outcomes, including assessment tools, statistical significance, assessment time points, and corresponding ICF coding.
