# Supplementary material for: Game-Based Interventions for Health Promotion in Older Adults: A Systematic Review of Protocols and ICF-Based Outcomes
Source: Healthcare (Basel). 2026 Jul 31;14(15):2313. doi: 10.3390/healthcare14152313 (PMC13465205; doi:10.3390/healthcare14152313)
Supplement: Supplementary file 1 [file healthcare-14-02313-s001.zip › S1_Database Specific Search Strategies.pdf]

Supplementary Material S1

Database-Specific Search Strategies

The following database-specific search strategies summarize the core search structure used to identify studies related to game-based interventions for health promotion in older adults.

PubMed
(("Aged"[Mesh] OR elderly OR "older adults" OR ageing OR aging)
AND
("Health Promotion"[Mesh] OR "health promotion" OR "health education")
AND
(game* OR gaming OR gamification OR "serious games" OR exergam*))
Filters applied: English and Portuguese language; publication years 2014–2026. The final search was conducted on 31 May 2026.

Scopus
TITLE-ABS-KEY (elderly OR "older adults" OR ageing OR aging)
AND TITLE-ABS-KEY ("health promotion" OR "health education")
AND TITLE-ABS-KEY (game* OR gaming OR gamification OR "serious games" OR exergam*)
Filters applied: English and Portuguese language; publication years 2014–2026. The final search was conducted on 31 May 2026..

Web of Science
TS=(elderly OR "older adults" OR ageing OR aging)
AND TS=("health promotion" OR "health education")
AND TS=(game* OR gaming OR gamification OR "serious games" OR exergam*)
Filters applied: English and Portuguese language; publication years 2014–2026. The final search was conducted on 31 May 2026.
