# Supplementary material for: Game-Based Interventions for Health Promotion in Older Adults: A Systematic Review of Protocols and ICF-Based Outcomes
Source: Healthcare (Basel). 2026 Jul 31;14(15):2313. doi: 10.3390/healthcare14152313 (PMC13465205; doi:10.3390/healthcare14152313)
Supplement: Supplementary file 1 [file healthcare-14-02313-s001.zip › Table S1_Methodological quality assessment of quasi-experimental studies using the Joanna Briggs Institute (JBI) critical appraisal checklist.pdf]

Table S1. Methodological quality assessment of quasi-experimental studies using the Joanna Briggs Institute (JBI) critical appraisal checklist.

Study (Author, year)	Q1	Q2	Q3	Q4	Q5	Q6	Q7	Q8	Q9
**Bao et al., 2025**	Y	Y	N	Y	Y	Y	Y	U	Y
**Berman et al., 2020**	Y	N	NA	N	Y	Y	U	Y	Y
**Chao et al., 2015**	Y	Y	Y	N	Y	Y	Y	Y	Y
**Clifford et al., 2014**	Y	N	NA	N	Y	Y	Y	Y	Y
**Hsu I et al., 2023**	Y	N	NA	N	Y	Y	Y	Y	Y
**Lee et al., 2014**	Y	N	NA	N	Y	Y	Y	Y	Y
**Lee et al., 2020**	Y	Y	Y	N	N	Y	Y	Y	Y
**Lee et al., 2015**	Y	N	NA	N	Y	Y	Y	Y	Y
**Martins et al., 2020**	Y	Y	Y	N	Y	Y	Y	Y	Y
**Mouton et al., 2017**	Y	Y	Y	N	Y	Y	Y	Y	Y
**Onishi et al., 2022**	Y	N	NA	N	Y	Y	Y	Y	Y
**Roopchand-Martin et al., 2015**	Y	N	NA	N	Y	Y	Y	U	Y
**Strand et al., 2014**	Y	N	NA	N	Y	Y	Y	Y	Y
**Szanton et al., 2016**	Y	N	NA	N	Y	Y	Y	Y	N
**Tsai et al., 2024**	Y	Y	Y	N	Y	Y	Y	Y	Y
**Tsai et al., 2025**	Y	N	NA	NA	Y	Y	Y	Y	Y

Y = Yes; N = No; U = Unclear; NA = Not applicable.
