# Supplementary material for: Game-Based Interventions for Health Promotion in Older Adults: A Systematic Review of Protocols and ICF-Based Outcomes
Source: Healthcare (Basel). 2026 Jul 31;14(15):2313. doi: 10.3390/healthcare14152313 (PMC13465205; doi:10.3390/healthcare14152313)
Supplement: Supplementary file 1 [file healthcare-14-02313-s001.zip › Table S2_Methodological quality assessment of randomized controlled trials using the Joanna Briggs Institute (JBI) critical appraisal checklist.pdf]

Table S2. Methodological quality assessment of randomized controlled trials using the Joanna Briggs Institute (JBI) critical appraisal checklist.

Study (Author, year)	Q1	Q2	Q3	Q4	Q5	Q6	Q7	Q8	Q9	Q10	Q11	Q12	Q13
Belchior et al., 2019	U	Y	U	Y	Y	Y	Y	Y	Y	Y	Y	Y	Y
Crandall et al., 2019	N	N	Y	N	N	Y	U	Y	Y	Y	Y	Y	Y
Dispennette et al., 2019	N	Y	Y	NA	U	Y	U	NA	Y	Y	Y	Y	Y
Huang et al., 2025	Y	U	Y	N	N	Y	Y	Y	N	Y	Y	Y	
Hughes et al., 2014	Y	U	Y	U	U	Y	U	Y	Y	Y	Y	Y	Y
Iizuka et al., 2019	Y	Y	Y	N	U	Y	U	Y	Y	Y	Y	Y	Y
Kwan et al., 2024	Y	Y	Y	N	N	Y	Y	Y	Y	Y	Y	Y	Y
Nonino et al., 2018	Y	U	Y	U	N	Y	N	Y	Y	Y	Y	Y	Y
Phirom et al., 2020	N	U	Y	U	U	Y	Y	Y	Y	Y	Y	Y	Y
Sayar et al., 2023	U	U	Y	U	U	Y	U	Y	Y	U	Y	Y	U
Shake et al., 2018	N	Y	Y	Y	Y	Y	Y	Y	Y	Y	Y	Y	Y
Verghese et al., 2021	Y	Y	U	Y	Y	Y	Y	Y	Y	Y	Y	Y	Y
Yang et al., 2022	Y	Y	Y	Y	U	Y	Y	Y	Y	Y	Y	Y	Y

Y = Yes; N = No; U = Unclear; NA = Not applicable.
