# Supplementary material for: Game-Based Interventions for Health Promotion in Older Adults: A Systematic Review of Protocols and ICF-Based Outcomes
Source: Healthcare (Basel). 2026 Jul 31;14(15):2313. doi: 10.3390/healthcare14152313 (PMC13465205; doi:10.3390/healthcare14152313)
Supplement: Supplementary file 1 [file healthcare-14-02313-s001.zip › Table S3_Outcome-level synthesis of health-related outcomes^J including statistical significance and ICF coding^.pdf]

Table S3. Outcome-level synthesis of health-related outcomes, including statistical significance and corresponding ICF coding.

Study	Outcome Domain	Outcome Measure	Assessment Tool	Time Point	Statistical Significant (Y/N)	ICF Code
Bao et al., 2025	Exercise Intention	Exercise Intention<br>Intention to exercise in the future	Exercise Intention Scale	Post-intervention	Y	d570
	Fear of Falling	Fear of falling	Single-item Fear of Falling Measure	Post-intervention	Y	b152
	Emotional Well-being	Emotional well-being	Mental Health Continuum Short Form (MHC-SF) items	Post-intervention	Y	b152
Belchior et al., 2019	Game performance	Performance on the Crazy Taxi game	Crazy Taxi	Post-intervention and Follow-up	Y	d155<br>d9200
	Game performance	Performance on the InSight game	InSight	Post-intervention and Follow-up	Y	d155<br>d9200
	Visual Attention	Useful Field of View	UFOV (Rochester)	Follow-up	Y	b1561
	Visual Attention	Useful Field of View	UFOV (UAB)	Post-intervention and Follow-up	Y	b1561
	Visual Attention	Attentional Blink	Attentional Blink Test	Post-intervention and Follow-up	Y	b140
	Visual Attention	Multiple Object Tracking	MOT	Post-intervention	Y	b140
	Activities of Daily Living	Time IADL	Timed IADL	Follow-up	Y	d230
	Emotional Function	Depressive Symptoms	Geriatric Depression Scale	Follow-up	Y	b152
	Visuospatial Function	Block Design	WAIS Block Design	Post-intervention and Follow-up	N	b1565
	Visuospatial Function	Judgment of Line Orientation	JOLO	Post-intervention and Follow-up	N	b1565
	Visuospatial Function	Object Rotation	Object Rotation Test	Post-intervention and Follow-up	N	b1565
	Visuospatial Function	Mental Rotation	Mental Rotation Test	Post-intervention and Follow-up	N	b1565
	Memory	Verbal Learning	HVLT	Post-intervention and Follow-up	N	b144

	Emotional Function	Positive Affect	PANAS	Post-intervention and Follow-up	N	b152
	Emotional Function	Negative Affect	PANAS	Post-intervention and Follow-up	N	b152
Berman et al., 2020	Health Literacy	Knowledge about depression and ageing	Item: "Depression is a normal part of getting older"	Post-intervention	Y	d198
	Health Literacy	Knowledge about depression symptoms	Item: "Depression may cause you to want to sleep more"	Post-intervention	Y	d198
	Health Literacy	Knowledge about depression	Item: "Being sad and having depression is the exact same thing"	Post-intervention	N	d198
	Health Literacy	Stigma regarding depression	Item: "Only weak people suffer from depression"	Post-intervention	Y	b126
	Health Literacy	Help-seeking knowledge	Item: "Talking with a therapist, counselor, or social worker can help a depressed older adult feel better"	Post-intervention	N	d198
	Health Literacy	Knowledge of physical consequences of depression	Item: "Depression can cause other physical health problems"	Post-intervention	Y	d198
	Health Literacy	Knowledge of benefits of physical activity for mood	Item: "Physical activity can improve your mood"	Post-intervention	Y	d198
	Health Literacy	Overall depression literacy score	Total score (7-item questionnaire)	Post-intervention	Y	d198
Chao et al., 2015	Balance	Balance	Berg Balance Scale (BBS-14)	Post-intervention	Y	b2351
	Mobility	Functional mobility	Timed Up and Go Test (TUG)	Post-intervention	Y	b770<br>d420<br>d450
	Physical Function	Walking endurance	Six-Minute Walk Test (SMWT)	Post-intervention	N	b455<br>d450
	Emotional Function	Depressive symptoms	Geriatric Depression Scale (GDS-15)	Post-intervention	Y	b152
	Quality of Life	Physical health-related quality of life	SF-8 Physical Component Summary (PCS)	Post-intervention	N	NC

	Quality of Life	Mental health-related quality of life	SF-8 Mental Component Summary (MCS)	Post-intervention	N	b152
	Falls-related Self-efficacy	Fear of falling	Falls Efficacy Scale (FES)	Post-intervention	N	b152
	Self-efficacy	Confidence to exercise	Self-Efficacy for Exercise Scale (SEE)	Post-intervention	N	d570
Clifford et al., 2014	Health Literacy	Knowledge of symptom management for URTI	Antibiotic Knowledge Survey	Post-intervention	Y	d198
	Health Literacy	Knowledge of symptoms requiring antibiotic treatment	Antibiotic Knowledge Survey	Post-intervention	Y	d198
	Health Literacy	Knowledge of influenza/common cold etiology	Antibiotic Knowledge Survey	Post-intervention	Y	d198
	Health Literacy	Knowledge of antibiotic effectiveness	Antibiotic Knowledge Survey	Post-intervention	N	d198
	Health Literacy	Knowledge of antibiotic resistance	Antibiotic Knowledge Survey	Post-intervention	N	d198
	Health Literacy	Total knowledge score	Antibiotic Knowledge Survey	Post-intervention	N	d198
Crandall et al., 2019	Health Activation	Knowledge, skills and confidence for self-management of health	Patient Activation Measure (PAM-10)	Post-intervention	Y	d570
	Health Literacy	Knowledge of falls prevention and osteoarthritis	30-item Health Knowledge Test	Post-intervention	Y	d198
Dispennette et al., 2019	Quality of Life	Physical Health	WHOQOL-BREF Domain 1	Post-intervention	N	b1300 b280 d450 d510 d850
	Quality of Life	Psychological Health	WHOQOL-BREF Domain 2	Post-intervention	N	b152
	Quality of Life	Social Relations	WHOQOL-BREF Domain 3	Post-intervention	N	d7
	Emotional Function	Positive Affect	PANAS	Post-intervention	Y	b152
	Emotional Function	Negative Affect	PANAS	Post-intervention	N	b152
	Falls-related Self-efficacy	Fear of Falling	Falls Efficacy Scale (FES)	Post-intervention	N	b152
	Mobility	Functional Mobility	Timed Up and Go (TUG)	Post-intervention	N	b770 d420 d450

	Lower-body Strength	Sit-to-Stand Performance	30-Second Chair Stand Test	Post-intervention	N	b730<br>b740
	Balance	Static Balance	4-Stage Balance Test	Post-intervention	N	b2351
	Muscle Strength	Handgrip Strength	JAMAR Dynamometer	Post-intervention	Y	b730
	Health Literacy	Knowledge of falls prevention and quality of life	30-item Health Knowledge Quiz	Post-intervention	Y	d198
Huang et al., 2025	Cardiovascular Function	Resting heart rate	Senior Fitness Test	Post-intervention	N	b4100
	Physical Activity	Total physical activity (Total MET)	IPAQ	Post-intervention	Y	d570
	Physical Activity	Vigorous physical activity	IPAQ	Post-intervention	Y	d570
	Physical Activity	Moderate physical activity	IPAQ	Post-intervention	N	d570
	Physical Activity	Walking	IPAQ	Post-intervention	N	d450
	Sedentary Behaviour	Sitting (work)	IPAQ	Post-intervention	N	d415
	Sedentary Behaviour	Sitting (holiday)	IPAQ	Post-intervention	N	d415
	Muscle Strength	Upper limb muscular endurance	Arm Curl Test	Post-intervention	Y	b730<br>b740
	Muscle Strength	Lower limb muscular endurance	Chair Stand Test	Post-intervention	Y	b730<br>b740
	Flexibility	Lower limb flexibility	Chair Sit-and-Reach Test	Post-intervention	N	b710
	Flexibility	Upper limb flexibility	Back Scratch Test	Post-intervention	N	b710
	Balance	Static balance	One-Leg Standing Test	Post-intervention	N	b2351
	Balance	Dynamic balance	8-Foot Up-and-Go Test	Post-intervention	Y	d450
	Cardiorespiratory Fitness	Aerobic endurance	2-Minute Step Test	Post-intervention	Y	b455
	Muscle Power	Lower body explosiveness	AFAscan Vertical Jump Test	Post-intervention	N	b730
	Core Strength	Core strength	AFAscan Sit-Up Test	Post-intervention	Y	b730<br>b740
	Flexibility	Flexibility	AFAscan Toe-Touch Test	Post-intervention	Y	b710
	Agility	Agility	AFAscan Agility Test	Post-intervention	Y	b760
	Balance	Balance	AFAscan Balance Test	Post-intervention	Y	b2351
	Endurance	Endurance	AFAscan Endurance Test	Post-intervention	Y	b455
	Body Composition	Body composition	InBody 270	Post-intervention	N	NC
	Quality of Life	Physical health domain	WHOQOL-BREF	Post-intervention	Y	b1300<br>b280<br>d450

						d510<br>d850
	Quality of Life	Psychological health domain	WHOQOL-BREF	Post-intervention	Y	b152
	Quality of Life	Social relationships domain	WHOQOL-BREF	Post-intervention	Y	d7
	Quality of Life	Environmental domain	WHOQOL-BREF	Post-intervention	N	e5
Hughes et al., 2014	Global Cognition	Cognitive performance	CAMCI Total	Post-intervention	N	b110
	Attention	Tracking A	CAMCI	Post-intervention	N	b140
	Executive Function	Tracking B	CAMCI	Post-intervention	N	b164
	Subjective Cognition	Cognitive complaints	CSRQ Cognitive	Post-intervention	N	b144
	Social Functioning	Social functioning	CSRQ Social	Post-intervention	N	d7
	Everyday Functioning	IADL performance	TIADL	Post-intervention	N	d230
	Physical Functioning	Gait speed	6-m Walk Test	Post-intervention	N	d450
Hsu et al., 2023	Health Literacy	Health Literacy Score	6-item Health Literacy Questionnaire	Post-intervention	Y	d198
	Emotional Function	Positive Emotions	PANAS-SF Positive Affect	Post-intervention	Y	b152
	Emotional Function	Negative Emotions	PANAS-SF Negative Affect	Post-intervention	N	b152
	Quality of Life	Self-perceived Quality of Life	EQ-VAS	Post-intervention	Y	NC
Iizuka et al., 2019	Working Memory	Visual Memory Span	VMST	Post-intervention	Y	b144
	Working Memory	Visual Memory Span Backward	VMSB	Post-intervention	Y	b144
	Working Memory	Visual Memory Span Forward	VMSF	Post-intervention	N	b144
	Working Memory	Digit Span	DS	Post-intervention	N	b144
	Working Memory	Digit Span Forward	DSF	Post-intervention	N	b144
	Working Memory	Digit Span Backward	DSB	Post-intervention	N	b144
	Game Performance	Go playing ability	Original Go Test	Post-intervention	Y	d155<br>d9200
	Verbal Function	Logical Memory I	LM I	Post-intervention	N	b144
	Verbal Function	Logical Memory II	LM II	Post-intervention	N	b144
	Verbal Function	Letter Fluency	Lf-K/H	Post-intervention	N	b167
	Verbal Function	Category Fluency	CF-A/V	Post-intervention	N	b167
	Executive Function	TMT-A/B	TMT-A/B	Post-intervention	N	b140<br>b164
Kwan et al., 2024	Mediterranean Diet Knowledge	Diet knowledge	Mediterranean Diet Quiz	Post-intervention	N	d198
	Diet Adherence	Mediterranean diet adherence	MDS	Post-intervention	Y	d570
	Global Cognition	Cognitive function	MoCA	Post-intervention	Y	b110

	Physical Frailty	Frailty status	Fried Frailty Phenotype	Post-intervention	Y	NC
	Grip Strength	Handgrip strength	Jamar Dynamometer	Post-intervention	N	b730
	Walking Speed	Walking	6MWT	Post-intervention	Y	d450
	Memory	Retrieval memory	FOME Retrieval	Post-intervention	N	b144
	Memory	Delayed recall	FOME Delayed Recall	Post-intervention	N	b144
	Verbal Fluency	Verbal fluency	FOME Combined Verbal Fluency	Post-intervention	N	b167
	Body Composition	Fat mass	InBody 770	Post-intervention	N	NC
	Body Composition	Skeletal muscle mass	InBody 770	Post-intervention	N	NC
Lee et al., 2014	Gait	Gait Velocity	GAITRite	Post-intervention	Y	d450
	Gait	Stride Length	GAITRite	Post-intervention	Y	d450
	Gait	Cadence	GAITRite	Post-intervention	Y	d450
	Gait	Double Support Time	GAITRite	Post-intervention	Y	d450
	Gait	Swing Time	GAITRite	Post-intervention	Y	d450
	Gait Variability	Coefficient of Variation of Stride Length	GAITRite	Post-intervention	N	d450
	Gait Variability	Coefficient of Variation of Swing Time	GAITRite	Post-intervention	N	d450
	Falls-related Self-efficacy	Balance Confidence	Balance Efficacy Scale (BES)	Post-intervention	Y	b152
Lee et al., 2020	Emotional Function	Depressive Symptoms	Geriatric Depression Scale – Short Form (GDS-SF)	Post-intervention	Y	b152
Lee et al., 2015	Health Behaviors	Physical domain	Health Behavior Assessment Tool	Post-intervention	Y	d570
	Cardiovascular Function	Systolic blood pressure	Digital Blood Pressure Monitor	Post-intervention	Y	b420
	Cardiovascular Function	Diastolic blood pressure	Digital Blood Pressure Monitor	Post-intervention	N	b420
	Cardiovascular Function	Pulse rate	Digital Blood Pressure Monitor	Post-intervention	Y	b4100
	Lower-body Strength	Chair Stand Test	CST	Post-intervention	N	b730<br>b740
	Mobility	Timed Up and GO	TUG	Post-intervention	Y	b770<br>d420<br>d450
	Flexibility	Sit and Reach Test	SRT	Post-intervention	N	b710
Martins et al., 2020	Muscle Strength	Handgrip Strength	Jamar Dynamometer	Post-intervention	Y	b730
	Lower Limb Strength	Sit-to-Stand Performance	30s STS	Post-intervention	Y	b730

						b740
	Mobility	Functional Mobility	Timed Up and Go	Post-intervention	N	d450
	Dynamic Balance	Step Test	Step Test	Post-intervention	Y	b760
	Balance	Static Balance	4StageBTM	Post-intervention	Y	b2351
	Self-Efficacy	Exercise Self-Efficacy	SEE	Post-intervention	Y	d570
	Participation	Activities and Participation related to Mobility	PAPM	Post-intervention	Y	d7-d9
Mouton et al., 2017	Physical Activity	Steps per day	ActiGraph GT3X+	Post-intervention and Follow-up	Y	d570
	Physical Activity	Energy expenditure/day	ActiGraph GT3X+	Post-intervention and Follow-up	Y	b540
	Quality of Life	EQ-5D Index Score	EQ-5D	Post-intervention	N	b152
	Quality of Life	EQ-5D Index Score	EQ-5D	Follow-up	Y	b152
	Motivation for Physical Activity	Relative Autonomy Index	BREQ-2	Post-intervention and Follow-up	N	d570
	Balance and Gait	Tinetti Score	Tinetti Test	Post-intervention	N	b2351
	Balance and Gait	Tinetti Score	Tinetti Test	Follow-up	Y	b2351
	Physical Performance	SPPB Total Score	SPPB	Post-intervention and Follow-up	N	b2351<br>d450<br>b730
	Mobility	Timed Up and Go	TUG	Post-intervention and Follow-up	N	b770<br>d420<br>d450
	Muscle Strength	Knee Extensor and Flexor Strength	MicroFET2	Post-intervention and Follow-up	N	b7300
	Muscle Strength	Hip Extensor and Flexor Strength	MicroFET2	Post-intervention and Follow-up	N	b7300
	Muscle Strength	Ankle Extensor and Flexor Strength	MicroFET2	Post-intervention and Follow-up	Y	b7300
Nonino et al., 2018	Attention	Executive attention	Trail Test B	Post-intervention	Y	b140
	Mobility	Timed Up and Go	TUG	Post-intervention	Y	b770<br>d420<br>d450
	Life Satisfaction	My life is close to my ideal	Satisfaction with Life Scale	Post-intervention	Y	d6-d9

	Life Satisfaction	If I could live my life again, I would change almost nothing	Satisfaction with Life Scale	Post-intervention	Y	d6-d9
Onishi et al., 2022	Positive Mood	Vigor	POMS	Post-intervention	Y	b152<br>b1300
	Negative Mood	Fatigue	POMS	Post-intervention	Y	b1300
	Negative Mood	Tension	POMS	Post-intervention	Y	b152
	Negative Mood	Anger	POMS	Post-intervention	N	b152
	Negative Mood	Confusion	POMS	Post-intervention	N	b140
	Negative Mood	Depression	POMS	Post-intervention	N	b152
	Cardiovascular Response	Heart Rate	Polar OH1	During/Post-intervention	Y	b4100
	Cardiovascular Response	Systolic Blood Pressure	BP Monitor	Post-intervention	N	b420
	Cardiovascular Response	Diastolic Blood Pressure	BP Monitor	Post-intervention	N	b420
Phirom et al., 2020	Fall Risk	Physiological fall risk	PPA Composite Score	Post-intervention	Y	b210<br>b260<br>b730<br>b750
	Balance	Postural sway	PPA	Post-intervention	Y	b750
	Reaction Time	Hand reaction time	PPA	Post-intervention	Y	b1402
	Vision	Contrast sensitivity	PPA	Post-intervention	N	b210
	Proprioception	Knee proprioception	PPA	Post-intervention	N	b260
	Muscle Strength	Knee extensor strength	PPA	Post-intervention	N	b730
	Mobility	Functional mobility	TUG single task	Post-intervention	N	b770<br>d420<br>d450
	Dual-task Mobility	Functional mobility under dual-task condition	TUG dual task	Post-intervention	Y	b770<br>d420<br>d450
	Global Cognition	Global cognitive performance	MoCA total score	Post-intervention	Y	b110
	Executive Function	Executive function	MoCA executive subtest	Post-intervention	Y	b164
	Attention	Attention	MoCA attention subtest	Post-intervention	Y	b140
	Language	Language	MoCA language subtest	Post-intervention	Y	b167
	Abstraction	Conceptual thinking / abstraction	MoCA abstraction subtest	Post-intervention	Y	b164

	Memory	Memory	MoCA memory subtest	Post-intervention	N	b144
	Naming	Naming	MoCA naming subtest	Post-intervention	N	b167
	Orientation	Orientation	MoCA orientation subtest	Post-intervention	N	b114
Roopchand-Martin et al., 2015	Balance	Functional balance	Berg Balance Scale (BBS)	Post-intervention	Y	b2351
	Dynamic Balance	Forward reach	Multi Directional Reach Test (MDRT)	Post-intervention	Y	b760 b7101
	Dynamic Balance	Backward reach	Multi Directional Reach Test (MDRT)	Post-intervention	N	b760 b7101
	Dynamic Balance	Right reach	Multi Directional Reach Test (MDRT)	Post-intervention	Y	b760 b7101
	Dynamic Balance	Left reach	Multi Directional Reach Test (MDRT)	Post-intervention	Y	b760 b7101
	Dynamic Balance	SEBT Overall Reach Score (Left Leg)	Star Excursion Balance Test (SEBT)	Post-intervention	Y	b760 b2351
	Dynamic Balance	SEBT Overall Reach Score (Right Leg)	Star Excursion Balance Test (SEBT)	Post-intervention	Y	b760 b2351
	Dynamic Balance	SEBT Anterior (Left)	SEBT	Post-intervention	Y	b760 b2351
	Dynamic Balance	SEBT Anterior-Medial (Left)	SEBT	Post-intervention	Y	b760 b2351
	Dynamic Balance	SEBT Medial (Left)	SEBT	Post-intervention	Y	b760 b2351
	Dynamic Balance	SEBT Posterior-Medial (Left)	SEBT	Post-intervention	Y	b760 b2351
	Dynamic Balance	SEBT Posterior (Left)	SEBT	Post-intervention	N	b760 b2351
	Dynamic Balance	SEBT Posterior-Lateral (Left)	SEBT	Post-intervention	N	b760 b2351
	Dynamic Balance	SEBT Lateral (Left)	SEBT	Post-intervention	N	b760 b2351
	Dynamic Balance	SEBT Anterior-Lateral (Left)	SEBT	Post-intervention	Y	b760 b2351
	Dynamic Balance	SEBT Anterior (Right)	SEBT	Post-intervention	Y	b760 b2351
	Dynamic Balance	SEBT Anterior-Medial (Right)	SEBT	Post-intervention	Y	b760 b2351

	Dynamic Balance	SEBT Medial (Right)	SEBT	Post-intervention	Y	b760<br>b2351
	Dynamic Balance	SEBT Posterior-Medial (Right)	SEBT	Post-intervention	Y	b760<br>b2351
	Dynamic Balance	SEBT Posterior (Right)	SEBT	Post-intervention	Y	b760<br>b2351
	Dynamic Balance	SEBT Posterior-Lateral (Right)	SEBT	Post-intervention	Y	b760<br>b2351
	Dynamic Balance	SEBT Lateral (Right)	SEBT	Post-intervention	Y	b760<br>b2351
	Dynamic Balance	SEBT Anterior-Lateral (Right)	SEBT	Post-intervention	N	b760<br>b2351
	Sensory Integration for Balance	Eyes Open Firm Surface	MCTSIB	Post-intervention	N	b2351
	Sensory Integration for Balance	Eyes Closed Firm Surface	MCTSIB	Post-intervention	N	b2351
	Sensory Integration for Balance	Eyes Open Foam Surface	MCTSIB	Post-intervention	N	b2351
	Sensory Integration for Balance	Eyes Closed Foam Surface	MCTSIB	Post-intervention	N	b2351
Sayar et al., 2023	Cardiovascular Response	Average Heart Rate	Polar H10	During intervention	Y	b4100
	Cardiovascular Response	Peak Heart Rate	Polar H10	During intervention	Y	b4100
	Cardiovascular Response	Double Product	HR × SBP	During intervention	Y	b420<br>b4100
	Physical Activity Enjoyment	Enjoyment of activity	PACES	Post-intervention	Y	d920
	Perceived Exertion	Exercise-related fatigue/perceived effort	RPE Scale	Post-intervention	Y	b455
	Cardiovascular Response	Systolic Blood Pressure	Blood Pressure Monitor	Post-intervention	Y	b420
	Cardiovascular Response	Diastolic Blood Pressure	Blood Pressure Monitor	Post-intervention	Y	b420
	Exercise Intensity	Time in HR Zone 1	Polar H10	During intervention	N	b455

	Exercise Intensity	Time in HR Zone 2	Polar H10	During intervention	N	b455
	Exercise Intensity	Time in HR Zone 3	Polar H10	During intervention	N	b455
	Exercise Intensity	Time in HR Zone 4	Polar H10	During intervention	N	b455
	Exercise Intensity	Time in HR Zone 5	Polar H10	During intervention	N	b455
Shake et al., 2018	Upper Body Strength	Upper body muscular strength	Arm Curl Test	Post-intervention	Y	b730
	Lower Body Strength	Lower body muscular strength and power	Repeated Chair Stands	Post-intervention	Y	b730<br>b740
	Gait Speed	Walking speed	4 Meter Walk	Post-intervention	N	d450
	Executive Function (Updating)	Working memory updating	Dot Counting (EXAMINER Battery)	Post-intervention	Y	b164
	Inhibitory Control	Response inhibition	Flanker Test	Post-intervention	N	b164
	Cognitive Flexibility	Set Shifting	Set Shifting Task	Post-intervention	N	b164
	Inhibitory Control	Response inhibition	Anti-Saccade Task	Post-intervention	N	b164
	Health Knowledge	Osteoarthritis and Fall Risk Knowledge	Health Knowledge Test	Post-intervention	N	d198
	Verbal Fluency	Phonemic Fluency	EXAMINER Battery	Post-intervention	N	b167
	Verbal Fluency	Category Fluency	EXAMINER Battery	Post-intervention	N	b167
	Balance	Side-by-Side Stand	SPPB	Post-intervention	N	b2351
	Balance	Semi-Tandem Stand	SPPB	Post-intervention	N	b2351
	Balance	Tandem Stand	SPPB	Post-intervention	N	b2351
	Physical Function	Total Score	SPPB	Post-intervention	N	b2351<br>d450<br>b730
Strand et al., 2014	Physical Activity Participation	Regular physical activity participation	Stages of Change for Physical Activity Questionnaire	Post-intervention (Week 8)	N	d570
	Physical Activity Participation	Regular physical activity participation	Stages of Change for Physical Activity Questionnaire	Follow-up (Week 25)	Y	d570
Szanton et al., 2016	Balance	Balance score	SPPB	Post-intervention	NR	b2351
	Gait Speed	Gait speed	SPPB	Post-intervention	NR	d450
	Lower Limb Strength	Chair stands	SPPB	Post-intervention	NR	b730<br>b740

	Physical Function	Total score	SPPB	Post-intervention	NR	b2351<br>d450<br>b730
	Quality of Life	Health-related quality of life	EuroQol	Post-intervention	NR	b152
Tsai et al., 2024	Osteoporosis Knowledge	Knowledge of osteoporosis	Osteoporosis Knowledge Scale	Post-intervention	Y	d198
	Osteoporosis Attitudes	Attitudes toward osteoporosis prevention	Osteoporosis Attitudes Scale	Post-intervention	Y	b126
	Osteoporosis Preventive Behaviors	Preventive behaviors related to osteoporosis	Osteoporosis Preventive Behaviors Scale	Post-intervention	Y	d570
Tsai et al., 2025	Dietary knowledge	Diabetes-prevention dietary knowledge	Dietary Knowledge Scale for Preventing Diabetes	Post-intervention	Y	d198
	Dietary Attitude	Attitudes toward diabetes-prevention diet	Dietary Attitude Scale	Post-intervention	Y	b126
	Dietary behavioural self-efficacy	Behavioral Self-Efficacy Confidence in performing healthy dietary behaviors	Dietary Behavioral Self-Efficacy Scale	Post-intervention	Y	d570
Verghese et al., 2021	Walking Speed	Normal walking speed	GAITRite	Post-intervention	N	d450
	Dual-task Mobility	Walking while talking speed	GAITRite	Post-intervention	Y	d450
	Physical Function	Total score	SPPB	Post-intervention	N	b2351<br>d450<br>b730
	Executive Function	Cognitive flexibility	Trail Making Test B	Post-intervention	N	b164
	Executive Function	Verbal fluency	Letter Fluency Test	Post-intervention	N	b167
	Processing Speed	Processing speed	Digit Symbol Substitution Test	Post-intervention	N	b147
Yang et al., 2022	Global Cognition	Cognitive functioning	SLUMSE	Post-intervention	N	b110
	Memory	Immediate recall	Contextual Memory Test (CMT)	Post-intervention	Y	b144
	Memory	Delayed recall	Contextual Memory Test (CMT)	Post-intervention	Y	b144
	Attention / Processing Speed	Trail Making Test A	TMT-A	Post-intervention	N	b140
	Instrumental Activities of Daily Living	Total IADL score	Lawton IADL Scale	Post-intervention	N	d230
	Telephone Use	Using the telephone	Lawton IADL Scale	Post-intervention	Y	d360
	Shopping	Shopping	Lawton IADL Scale	Post-intervention	N	d6200

	Food Preparation	Preparing food	Lawton IADL Scale	Post-intervention	N	d630
	Housekeeping	Housekeeping	Lawton IADL Scale	Post-intervention	N	d640
	Laundry	Doing laundry	Lawton IADL Scale	Post-intervention	N	d640
	Transportation	Using transportation	Lawton IADL Scale	Post-intervention	N	d470
	Medication Management	Handling medications	Lawton IADL Scale	Post-intervention	N	d570
	Financial Management	Handling finances	Lawton IADL Scale	Post-intervention	N	d860

Y- yes; N- No; NR – outcome assessed but statistical significance was not reported; NC – Non classification
